# Impact of chewing muscle anesthesia on masticatory performance in healthy participants

**DOI:** 10.1038/s41598-025-13580-5

**Published:** 2025-09-12

**Authors:** Dyanne Medina Flores, Tilda Karlsson, Giancarlo De La Torre Canales, Natalie Kaspo, Clara Malmström, Sara Messerer, Abhishek Kumar, Johanna Svedenlöf, Paulo Cesar Rodrigues Conti, Nikolaos Christidis, Anastasios Grigoriadis

**Affiliations:** 1 Division of Oral Rehabilitation, Department of Dental Medicine, Karolinska Institutet, Huddinge, and Scandinavian Center for Orofacial Neurosciences, Huddinge, Sweden; 2 Department of Prosthodontics and Periodontics, Bauru School of Dentistry, University of São Paulo (FOB/USP), Bauru, SP Brazil; 3 Egas Moniz Center for Interdisciplinary Research (CiiEM), Egas Moniz School of Health and Science, Almada, Portugal

**Keywords:** Chewing, Masticatory efficiency, Masticatory ability, Fatigue, Orofacial pain, Muscle

## Abstract

The impact of the muscle spindles in the masticatory performance remains elucidated. This study aimed to investigate the impact of local anesthesia in the jaw-closing muscles on masticatory performance in healthy participants. Thirty healthy pain-free volunteers underwent in two rounds of chewing tasks involving two types of viscoelastic candies and a two-coloured chewing gum. Lidocaine (3.0 mL) was injected into a total of six points in the masseter and temporalis muscles bilaterally for the second round. Pain intensity, fatigue, the number and area of particles, and the degree of mixing of the chewing gum were assessed at baseline and after each round of chewing. The number of candy particles after injection of lidocaine, was significantly lower (33%) for women compared to the results without anesthesia (*p* < 0.05). There was no significant difference in the variance of hue of the two-coloured chewing-gum when comparing values before and after anesthesia. None of the participants experienced any pain during the experiment. However, self-reported fatigue increased during the second round, i.e., after anesthesia, with significantly higher values observed at the final assessment point. (*p* = 0.029). Local anesthesia of the jaw-closing muscles appears to impair the masticatory function in women, leading to reduced efficiency in food comminution compared to normal mastication. The observed sex differences suggest that women may be more vulnerable to neuromuscular control alterations following sensory alterations.

## Introduction

Mastication is a complex process involving facial muscles, salivary secretion, teeth, and numerous receptors in the orofacial region, all of which have crucial connections to several areas of the brain^1^. The rhythmic mandibular movements involved in mastication are generated by the masticatory muscles, which primarily consist of the temporalis, masseter, medial pterygoid and lateral pterygoid muscle^2^. This is made possible by the innervation from the mandibular branch of the trigeminal nerve through alpha-motoneurons that are located in the trigeminal motor nuclei within the brainstem^3^.

The movements during mastication enable the comminuting of food by opening the mouth and occluding with the precise force needed to bring the teeth into intermittent contact^4,5^. This precision is possible due to the continuous sensory feedback, primarily from periodontal mechanoreceptors and muscle spindles, which allows continuous adaptation of muscle activity based on the properties of the food throughout the masticatory sequence^5–7^. In addition to this, it has been shown that mechanoreceptors in the temporomandibular joint, facial skin, lips, oral mucosa, and dental pulp have also been shown to provide important proprioceptive information during mastication^8–10^.

Masticatory muscles are richly innervated by muscle spindles, with the majority being found in the temporalis and masseter muscles^11,12^. Muscle spindles are highly complex and sensitive sensory receptors distributed throughout the belly of the muscles, that provide precise information about proprioception as well as changes in length and velocity of contraction in muscle length and velocity^13^. They are composed of several intrafusal fibers innervated by alpha, gamma and beta motor neurons, whose cell bodies in the mesencephalic nucleus of the trigeminal nerve^13^. It has been demonstrated that muscle spindles in limb muscles are important for maintaining static posture based on their proximity to fatigue resistant motor units that generate small forces^14^. Input from muscle spindles in the masseter muscle, however, appears to be more effective on larger motor neurons, suggesting that they may rather be important for load compensation and the generation of large bite forces^14^. Studies show that the firing frequency of the muscle spindles during mastication is based on the hardness of the food and in correlation with jaw muscle activity, leading to the conclusion that muscle force regulation during mastication is mediated by muscle spindles^6,7,15,16^.

Masticatory performance is commonly evaluated by assessing the ability to comminute food based on particle size distribution or surface area of food particles^17–20^. Previous research has objectively examined jaw kinematics and masticatory function in various populations, including older adults, denture wearers, dental implant users, and individuals with chronic jaw myalgia^21–26^. Other studies have demonstrated that as food hardness increases, muscle activity in healthy, pain-free jaw-closing muscles increases, primarily due to sensory feedback from periodontal receptors and spindle afferents^5,6,27,28^ . Although few studies have addressed the impact of nociceptive influences on jaw muscle proprioception^29^, to our knowledge, no study has directly assessed masticatory function by selective reduction of these inputs. Understanding how muscle spindle input contributes to chewing efficiency is essential for refining diagnostic and therapeutic strategies for conditions affecting jaw function.

Furthermore, individuals experiencing impaired mastication may modify their food choices or swallow larger pieces as a compensatory strategy during eating^1^. Unbalanced food choices, such as favoring softer and more easily consumed products instead of hard-to-chew food can lead to imbalanced dietary intake, while swallowing larger pieces may lead to gastrointestinal disturbances due to reduced nutrient bioavailability^1^. Thus, an efficient masticatory function is considered fundamental to supporting overall general health and quality of life in humans^7^.

To effectively address symptoms of impaired masticatory function, it is crucial to understand the full range of mechanisms involved in the masticatory system. In addition, there is a lack of studies evaluating masticatory function following reduced muscle spindle input in humans, highlighting the need to determine the role of muscle on masticatory performance. Given these considerations, the present study aims to investigate how masticatory performance is affected by anesthetizing the jaw closing muscles in young healthy participants. The working hypothesis is that altering muscle afferents via anesthesia of the muscles impairs the ability to finely commute food, while mixing ability remains unaffected due to compensatory function of the tongue.

## Material and methods

### Participants

Thirty healthy and pain-free participants (15 men and 15 women) aged between 18 and 40 years were enrolled in this study. The sample size calculation indicated that a minimum of 12 participants were needed to achieve a power of 80%, a significance level of *p* < 0.05 and an effect size of 1.2^26^; to account for potential missing data and participant dropout, 15 participants were included in each group. They were divided into two groups based on sex in order to subsequently evaluate sex differences. The volunteers were recruited at the University Dental Clinic of the Department of Dental Medicine, Karolinska Institutet, Huddinge, Sweden. The study was conducted in accordance with the Declaration of Helsinki and approved by the Regional Ethical Review Board in Stockholm (DNR: 2014/1394-3). Written informed consent was obtained from all participants.

Inclusion criteria for all participants were (a) of good general health with no known diseases or regular intake of medication; (b) age between 18 and 40. Exclusion criteria were (a) signs of TMD according to DC/TMD; (b) bridges or implants in premolar or molar region; (c) two or more crowns on molars; (d) class II or III malocclusion; (e) current dental problems; (f) tobacco use 7 days prior to experiment; (g) NSAID intake 2 days prior to experiment; (h) pregnancy or recent delivery; (i) allergy to Lidocaine; (j) no use of hormonal contraceptives i.e. pills, implant, patch or hormonal IUD.

### Experimental protocol

Prior to inclusion, to make sure that all inclusion criteria were met, digital questionnaires regarding pain, jaw function, oral health, fear of movement, physical symptoms, psychosocial signs, and masticatory ability were completed by all participants. In addition, a brief clinical examination of the participant according to Axis I of DC/TMD was performed^30^. All participants were then asked to complete two rounds of chewing tasks involving two soft elastic candies, two hard plastic candies, and one pair of two-coloured gum. During each task, chewing duration was measured and chewing strokes were counted. Before starting the second round, local anesthesia was injected bilaterally into the masseter and temporalis muscle. Pain intensity and self-reported fatigue were assessed at baseline and after every task, before and after the local anesthesia. The candies were weighed in grams (g) both before and after the experiment. One examiner (TK) provided comprehensive information about the experiment, conducted the initial clinical examination as well as assessed pain and fatigue between each task. The other examiner (NK) provided instructions before each task, monitored the time and number of chewing strokes, and delivered the local anesthesia before round two.

### Psychosocial questionnaires

The questionnaires included were: Graded chronic pain scale (GCPS)^31^, Oral behavior checklist (OBC-21)^32^, Jaw functional limitation scale (JFLS-20)^33^, Pain catastrophizing scale (PCS-13), Perceived stress scale (PSS-10), Oral health impact profile (OHIP-5), Tampa scale of kinesiophobia (TSK-TMD)^34^, The patient health questionnaire for depression (PHQ-9)^35^, The patient health questionnaire for physical symptoms (PHQ-15)^36^ and Generalized anxiety disorder scale (GAD-7)^37^.

### Test food

The mixing ability was examined using a standardized two-coloured gum called Vivident Fruitswing® “Karpuz/Asai Üzümü” (Vivident) (Perfetti van Melle). This gum consists of two inseparable layers, violet and green, and is a reliable and widely accepted method used in several previous studies^26,38^. To examine the comminuting ability, viscoelastic candies were utilized^21,28,39^. Red, round, sugar-coated sour gummy gelatin candies (Haribo Syrlingar, Haribo Lakrits AB) served as hard plastic candy, while large, red, heart-shaped jelly candies (Stora Gelé Hjärtan, Konfektyrfabriken Aroma AB) served as soft elastic candy^26,40^. One examiner (TK) manually cut and shaped all the soft elastic candies into cubes measuring the standardized dimensions of 20 × 20 × 5 mm, while the round-shaped hard candies already met the standardized dimensions of 20 × 5 mm.

### Chewing tasks

**Fig. 1:**
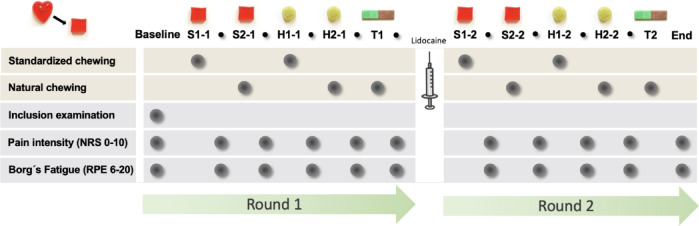
Flowchart of the experiment. Experimental protocol with sequence of assessments and chewing tasks for each candy and chewing gum (S1-1, S2-1, H1-1, H2-1, T1, S1-2, S2-2, H1-2, H2-2, T2). The candies are named after the type of candy with the first letter (S = soft, H = hard), type of chewing task with the first number (1 = standardized chewing, 2 = natural chewing) and which round with the second number (1 = round one, 2 = round two). The two coloured chewing gums are named T1 for round one and T2 for round two. Pain intensity and self-reported fatigue were assessed at baseline and after every task.

The participants performed two identical rounds of chewing tasks where they chewed two pieces of soft candies (S1 and S2), two pieces of hard candies (H1 and H2) and one two-coloured gum. Participants were instructed to begin with S1, chewing the candy in a fixed number of cycles, 20 times using their preferred side and then spitting it out along with their saliva into a paper cup. Next, they were instructed to chew S2 as naturally as possible until swallowing threshold, while the examiner (NK) counted every chewing stroke. Instead of swallowing, they spat the chewed candy and saliva into a paper cup for later spreading on a transparent sheet. The same tasks were followed for the hard candies, H1 was chewed 20 times and H2 until swallowing threshold. All the paper cups containing the chewed candies were marked and saved for subsequent spreading on transparent sheets. Additionally, to assess the mixing ability, participants were instructed to chew the two-coloured gum for fixed number of 20 cycles, following the manual of Schimmel^38^ . They were asked to chew as naturally as possible, allowing them to change chewing side during the strokes, and then spit it out. After every task, the participants were told to rinse their mouths with water. A second round of chewing tasks, identical to the first one, was then performed after injection of local anesthesia into the masseter and temporalis muscles. The test sequence was not randomized, based on methodological considerations and experimental feasibility. See flowchart in Fig. 1.

### Local anesthesia

Lidocaine (Lidocaine Hydrochloride 10 mg/ml, Mylan Ireland Limited) was administered as a local anesthetic, with a total dose of 3 ml injected^41,43^. Thus, 0,5 ml was delivered at three points: one in the temporalis muscle (approximately 2 cm posterior to the outer corner of the eye) and two in the masseter muscle (approximately 1 cm anterior to the ear and below the zygomatic arch, into the posterior part of the masseter and in the belly of the masseter, approximately 1,5 cm above the mandible’s lower border).

### Assessment of pain and fatigue

Pain intensity and self-reported fatigue were assessed at baseline and after each chewing task in both rounds. Fatigue was assessed using Borg’s Rating of Perceived Exertion (Borg’s RPE) Scale. Participants were asked to rate their perceived exertion on this scale during the tasks, where a rating of 6 corresponds to extremely easy effort and a rating of 20 indicates maximal effort^44^. Pain was measured using a numeric rating scale (NRS) from 0 to10 where 0 = no pain and 10 = worst imaginable pain^45^.

### Imaging, pre-processing and data analyses

After both rounds of chewing tasks were performed, all the fragmented candies in the paper cups were manually spread out on a transparent sheet with standardized dimensions of 100 mm × 100 mm. The candies were allowed to dry until the next day and scanned within the next 24 h. The chewed two-coloured gum was immediately put in a plastic bag with standardized dimensions of 60 mm × 90 mm × 1 mm and was flattened for scanning within 24 h. The candies, paper cups and the transparent sheets that were going to be used were weighed in grams (g) before the experiment. The next day the dried fragmented candies were weighed again. The scale used throughout this experiment was a digital gram scale with a precision of ± 1.0 g (Salter ARC 1066 BKDR15; Tonbridge, UK)^26,46^.

**Fig. 2:**
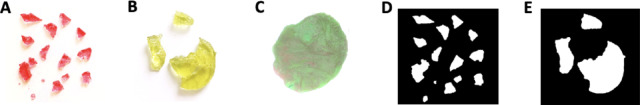
Ilustration samples of fragmented soft candy (**A**), hard candy (**B**), two coloured gum after chewing (**C**) and pictures to illustrate the processed image before analysis (**D**, **E**).

The scanning device Ricoh eduPrint Scanner was used to scan the samples of the fragmented candies and flattened chewing gum. All the samples were scanned with a white paper background. This device provides the possibility to image a two-dimensional illustration of the candies and chewing gum which were standardized with the following settings: Color → Original type-full color, Resolution → 300 dpi, Scanning-format → Cropped scanning in mm 210 × 297/5 × 5/70 × 70 for the chewing gum and 210 × 297/5 × 5/100 × 100 for the candy samples and Reproduction ratio → 100%. A standardized setting by Adobe Photoshop CC software (version 19.1.3, Adobe Systems Incorporated, San Jose, CA, USA) was used to preprocess the candy images to remove any shadows Fig. 2

Subsequently, candies were then analyzed with a standardized setting by Fiji Image J (Image Processing and Analysis in Java; National Institutes of Health, USA). The settings in Fiji Image J were as follows: Scale → width: 1200, height: 1200, distance in pixels: 11.4, known distance: 1.00, pixel aspect ratio: 1.0, unit of length: mm, Type → 8-bite, Size → width: 1185, height: 1185, depth: 1, Color threshold → black and white: dark background, Set measurements → area, Feret´s diameter and Analyze Particles → Size (mm): 0-infinity, circularity: 0–1, show: outlines, display results, clear results, summarize, add to manager, include holes. The results were presented in number of particles per original candy, the mean area of those particles in millimeters (mm) and the measurement of each in minimum Feret´s diameter due to their irregular shape. ^26^.

The ViewGum^©^, which is the Hue-Check Gum® own a validated analyzing software (freeware), was used to analyze the variance of hue (VOH) according to the instructions of the manual^38,47,48^.

### Statistical analyses

The statistical analyses were performed using SPSS software (IBM Corp. Released 2021. IBM SPSS Statistics for Windows, Version 28.0. Armonk, NY: IBM Corp). Normality of the data was tested with the Shapiro–Wilk test. Since all the variables showed a non-normal distribution the non-parametric Friedman test was used for intra-group comparisons, while the Mann Withney U test was employed for inter-group comparisons. All masticatory variables, pain and fatigue were presented as medians with the interquartile range (IQR). The significance level was set at *p* < 0.05.

## Results

The study comprised 30 participants, 15 healthy age-matched women and 15 healthy men with a median (min–max) age of 24.8 (20–30) with no significant differences between groups (*p* > 0.05). All the participants were asymptomatic, without any TMD symptoms according to DC/TMD (Table 1).

**Table 1:** Self-reported characteristics at baseline for 30 healthy participants.

Variable	Score
Age	24.7
Characteristic pain intensity (CPI)	
CPI	2.3
Grade 0	n = 25
Grade I	n = 5
Grade II	n = 0
Grade III	n = 0
Grade IV	n = 0
Oral behavior checklist (OBC-21)	21.4
Jaw functional limitation scale (JFLS-21)	
Chewing	0
Mobility	0
Communication	0
Total	0
Oral health impact profile (OHIP-5)	0.4
Tampa scale of kinesophobia (TSK-TMD)	21.4
Pain catastrophizing scale (PCS-13)	4.8
Perceived stress scale (PSS-10)	8.7
The patient health questionnaire for depression (PHQ-9)	3.1
The patient health questionnaire for physical symptoms (PHQ-15)	3.7
Generalized anxiety disorder scale (GAD-7)	3.4

Data are expressed as mean value for all variables. Age was assessed in years.

**Table 2:**
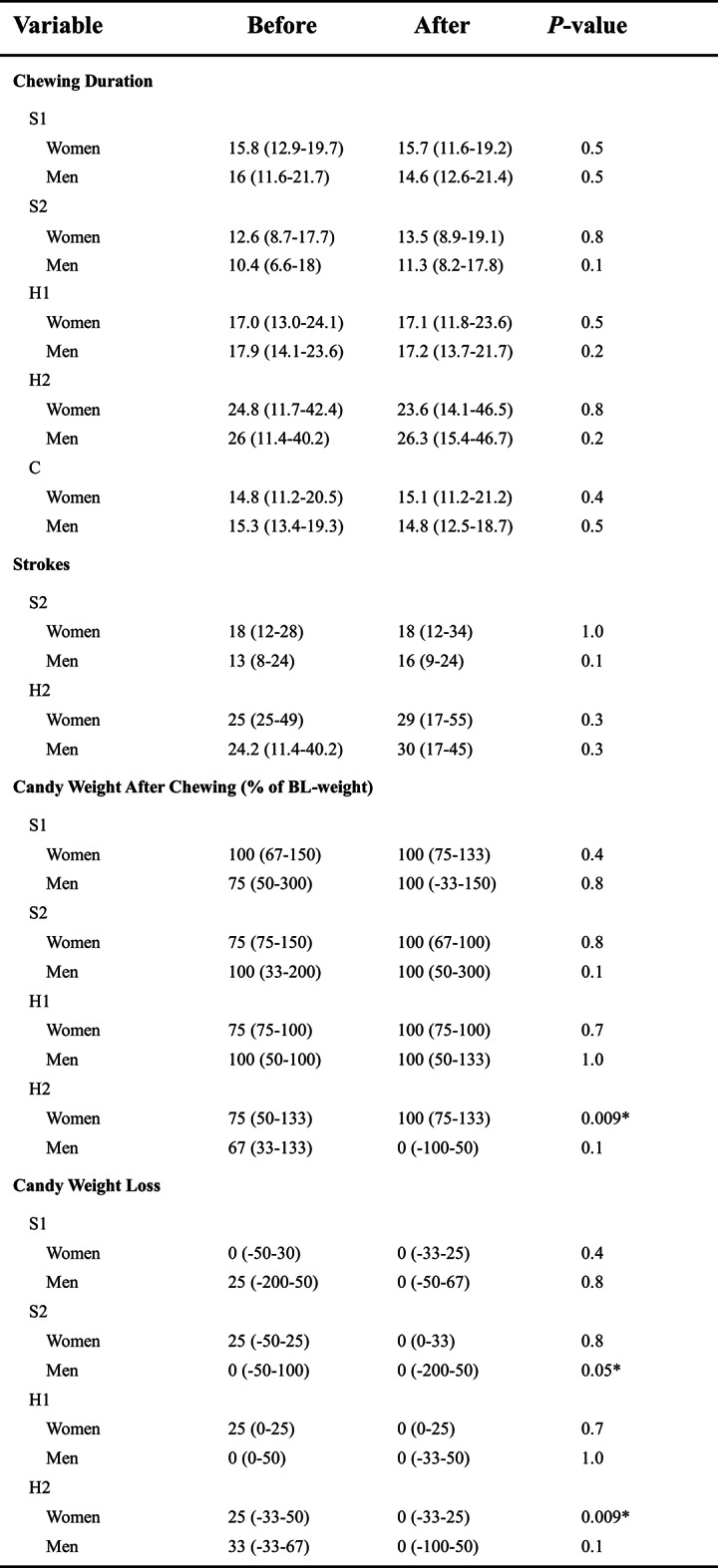
Clinical and experimental variables of chewing activity and efficiency measures before and after injection.

Data are expressed as median (min–max) for all variables. BL = Baseline. Chewing duration was assessed in seconds, weight loss in percent. *Significant difference *P* < 0.05.

**Table 3:**
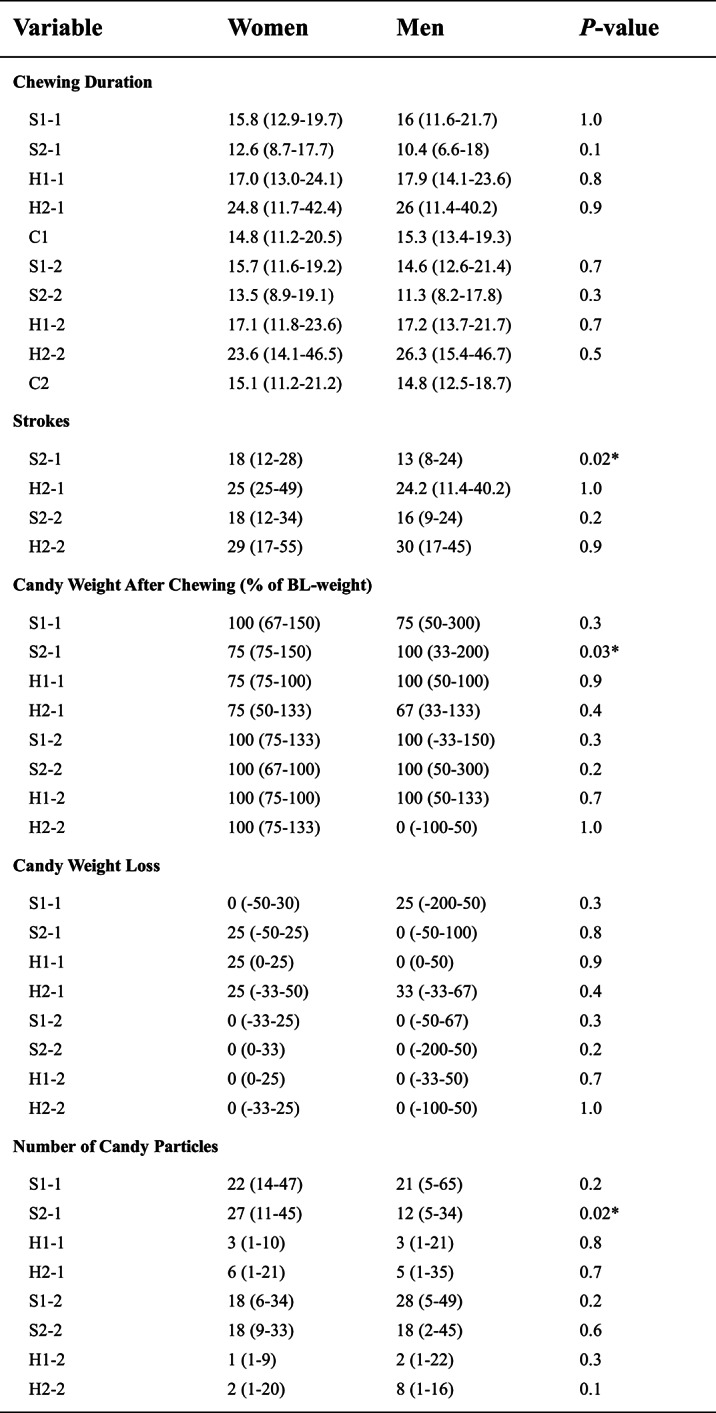
Clinical and experimental variables of comminution and mixing ability measures before and after injection (Number and size of candy particles, and hue of gum)

Data are expressed as median (min–max) for all variables. Area of particles in square millimeter, minimum Feret´s diameter in millimeter and hue of gum in variance. *Significant difference *P* < 0.05.

**Table 4:**
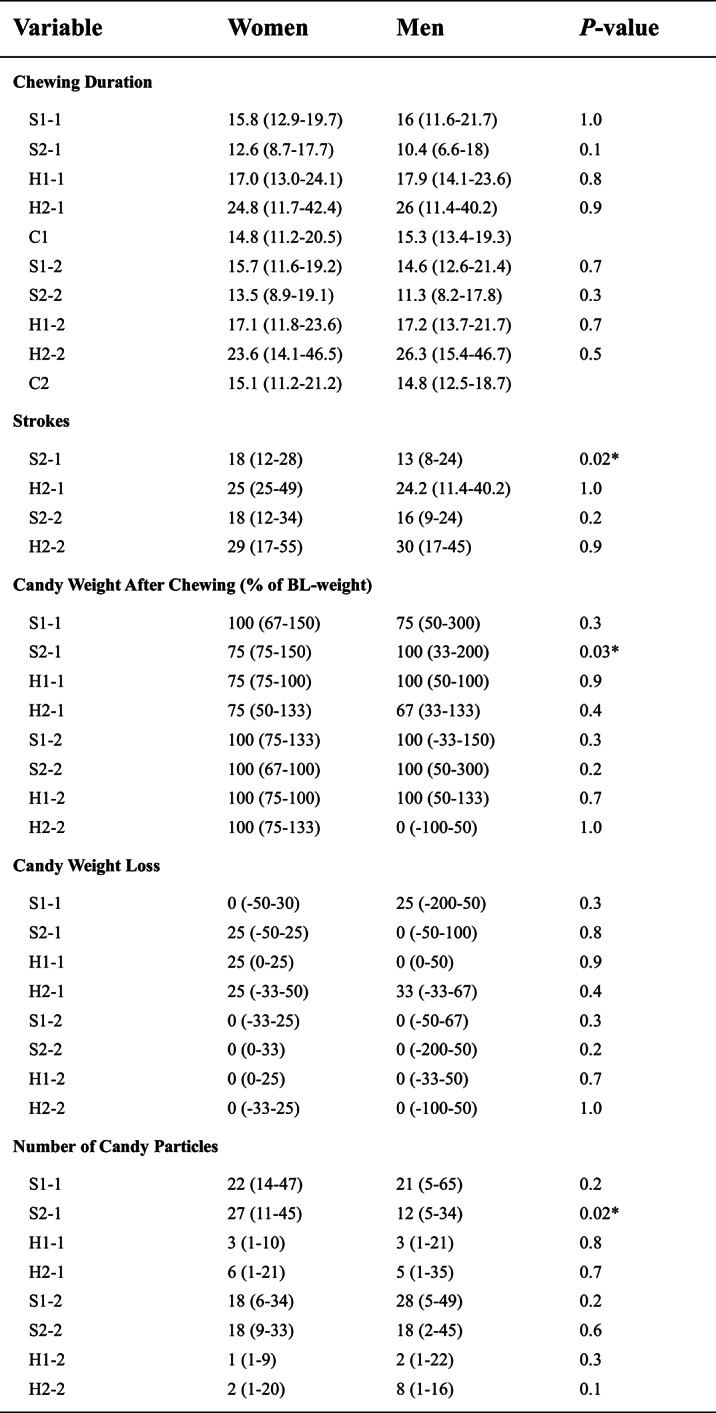
Clinical and experimental variables of chewing activity and efficiency measures comparing both groups: women and men.

Data are expressed as median (min–max) for all variables. BL = Baseline. Chewing duration was assessed in seconds, weight loss in percent. *Significant difference *P* < 0.05.

**Table 5:**
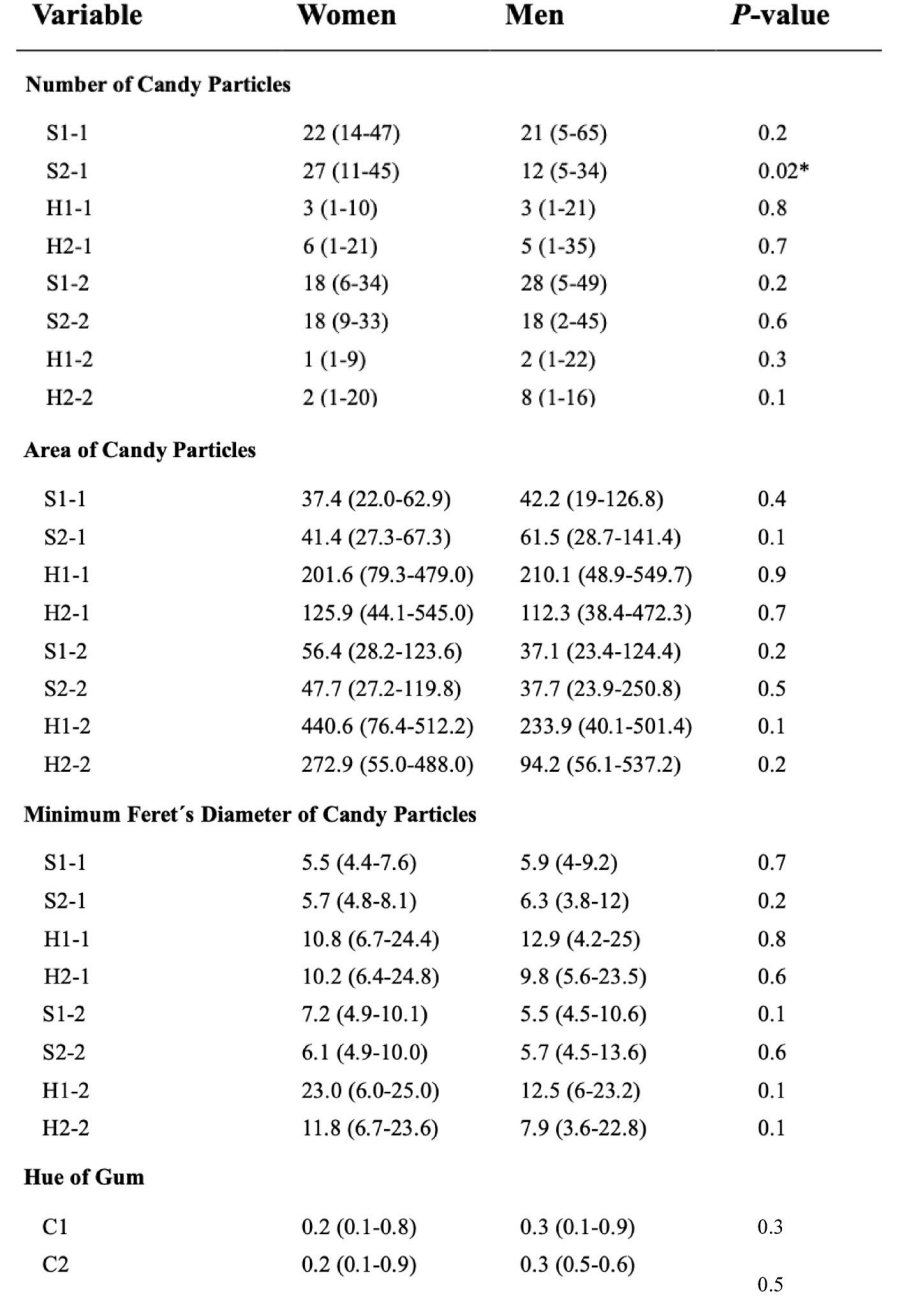
Clinical and experimental variables of comminution and mixing ability measures comparing both groups: women and men. (Number and size of candy particles, and hue of gum).

Data are expressed as median (min–max) for all variables. Area of particles in square millimeter, minimum Feret´s diameter in millimeter and hue of gum in variance. *Significant difference *P* < 0.05.

### Psychosocial questionnaires

The results showed low values for all the questionnaires used in both groups, indicating that the participants did not present any psychosocial distress or any orofacial pain that could have compromised the results (Table 1).

### Chewing performance

There were no significant differences (*p* > 0.05) in chewing duration and chewing strokes when comparing values before and after anesthesia for either soft candies, hard candies, or the two-coloured gum in women and men. (Table 2‚3). A statistically significant higher number of chewing strokes was observed for the soft candy S2-1 before injection between the groups (*p* < 0.02). (Table 4‚2).

Chewing tasks resulted in smaller weight loss in round two, but only the natural chewing task with hard candy H2 showed a significantly weight loss (*p* < 0.009) for women. However, the natural chewing task with soft candy S2 also showed a significant weight loss for men (*p* < 0.05) (Table 2). Significant differences were not found between groups. (Table 3).

The number of candy particles was significantly lower after anesthesia for soft candy S1 (*p* < 0.0001), soft candy S2 (*p* < 0.01), hard candy H1 (*p* < 0.03), and hard candy H2 (*p* < 0.04), in women. However, for men there were no differences in the number of candy particles for either soft or hard (Table 4). Comparisons between groups showed that only the number of candy particles of the soft candies S2-1 before injection was lower in men (*p* < 0.02) (Table 5).

The mean area of candy particles was larger in round two compared to round one for soft candy S1 (*p* < 0.001) and soft candy S2 (*p* < 0.001) in women. On the other hand, men did not present differences in the area of candy particles in any candy. (Table 4) Any significant differences were found between groups.

Regarding minimum Feret´s diameter, the standardized chewing task for soft candy (S1) and natural chewing task for hard candy (H2) exhibited a statistically significant larger Minimum Feret´s diameter after injection, with *p*-values of 0.001 and 0.01 respectively, as shown in Table 4. In contrast, men did not present any differences in the Minimum Feret’s diameter. No significant differences were found between groups.

Finally, there were no significant differences in the variance of hue of the two-coloured gum when comparing values before and after anesthesia or between sexes.

### Pain and fatigue

The pain intensity for all participants did not change at any assessment. In both women and men, the baseline value was 0, as well as for all individual tasks reporting pain with soft and hard candies. There was no difference in pain intensity in round one (before injection) when compared to those in round two (after injection) or between groups.

**Fig. 3:**
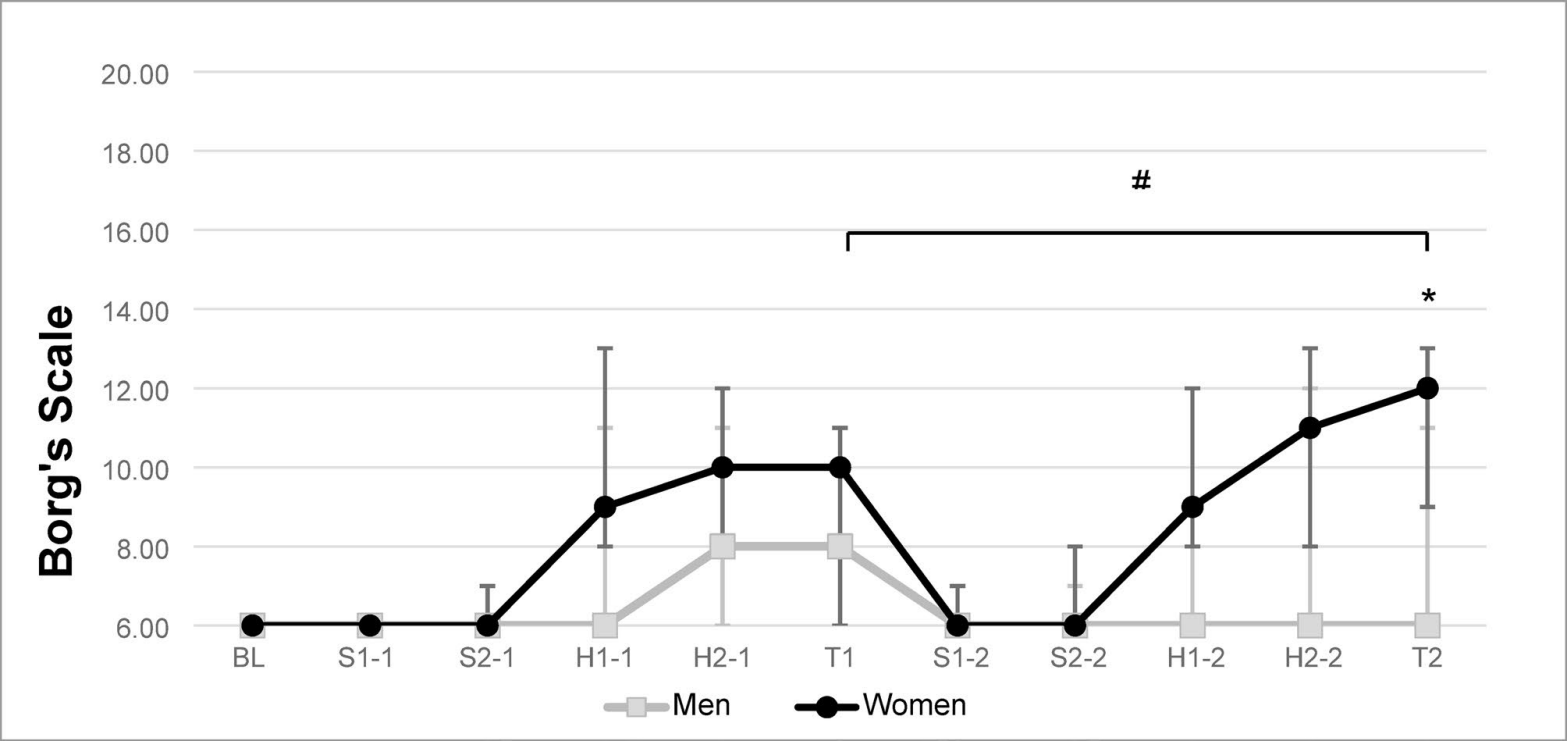
In this figure, the median is represented by the points on the line, the lower limit of the interval represents the first quartile (25%), while the upper limit represents the third quartile (75%). The width of the interval represents the interquartile range (IQR) of self-reported fatigue scores assessed with Borg’s rating of perceived exertion are shown at baseline and after chewing task S1, S2, H1, H2 and the two-coloured gum (T) before and after injection of local anesthetics for women and men. *Significant higher scores after injection comparing women and men (*p* < 0.026). # Significant higher scores after injection compared to before injection for women (Friedman ANOVA test; *p* < 0.029).

Higher self-reported fatigue was reported by women after injection with the two-coloured gum (*p* < 0.029), as shown in Fig. 3. Meanwhile, men reported fatigue only during the H2 hard candy and two-coloured gum (T) tasks before injection, without any statistically significant differences compared to after injection. Comparisons between groups showed higher fatigue values for women for the two-coloured gum (T) after injection (*p* < 0.026) (Fig. 3).

## Discussion

The findings of the present study demonstrated reduced masticatory performance and lower efficiency in comminuting food after receiving injections of local anesthetics in healthy participants. This decrease is likely due to diminished afferent signaling to the masseter and temporalis muscles. Masticatory performance, in this context, depends on the effective breakdown of food, which is assessed by the number of particles after chewing^46^. Based on our results, this seems to be influenced by gender, as anesthetizing the temporalis and masseter muscles did not appear to affect the chewing performance in men.

When comparing chewing duration patterns in both rounds, the participants used a shorter duration when naturally chewing the soft candy compared to the standardized chewing task. In contrast, for the hard candy, a longer chewing duration was preferred when chewing naturally, suggesting an adaptation to food hardness both before and after injection of local anesthetics. Since the anesthetic is expected to block muscle spindle input, it is possible that this adaptation is due to sensory input from periodontal mechanoreceptors^49,50^. This aligns with a previous study in which patients with chronic myalgia in the masseter muscles also displayed this adaptability to food hardness, despite impaired masticatory performance^26^.

The number of particles produced was the most distinct difference observed between round one and round two in women participants; they were able to chew the candy into significantly more particles before the injection compared to after. Given that the duration and number of chewing strokes remained nearly constant between the two rounds, this disparity likely arises from muscle force adaptation. When local anesthetics are injected into the masseter and temporalis muscles, they block sodium channels on the nerve cell membranes, leading to the inhibition of action potentials. This primarily inhibits pain and temperature perception by blocking Aδ and C fibers^51^. More relevant to this study, it also inhibits proprioception by blocking Aγ and Aβ fibers, thus blocking the input from muscle spindles^51^. Muscle spindles, together with periodontal mechanoreceptors, provide continuous sensory feedback essential for adapting chewing and muscle activity based on the food’s consistency^5–7^.

Some degree of this adaptation persisted for women participants even after the injection of local anesthetics on the second round, as indicated by the similar duration and number of chewing strokes required for both hard and soft candies. Despite this, participants struggled to break both candies into fine particles. The inhibition of muscle feedback seems to affect muscle force regulation, impairing the ability to apply the appropriate load needed to fragment the candies into small particles. This finding aligns with previous research suggesting that muscle spindles are crucial for muscle force regulation and the development of large bite forces^14–16^. Although muscle spindles play a significant role, other sensory receptors (such as mechanoreceptors in the periodontal ligaments, temporomandibular joint, and pulp) also provide essential proprioceptive information during mastication^8–10^. Despite the local injection of anesthesia into the muscles, it cannot be ruled out that these peripheral sensory receptors were also affected.

On the other hand, men didn’t show significant differences in the numbers of particles produced before and after injection. While it could be hypothesized that the muscle spindles were not sufficiently affected by the local anesthesia in male participants, due to the need of a larger dose of lidocaine for it to have its full effect considering that masseter muscle can be, thicker, wider and has a greater length in men than in women^52,53^. A more plausible explanation may lie in physiological differences related in neuromuscular control, as greater levels of muscular activity and greater occlusal force than those of females has been reported by researchers^54^. Rather than a suboptimal anesthetic effect, the preserved performance observed in men may be attributed to compensatory mechanisms involving the periodontal mechanoreceptors (PMRs). These receptors, which play a central role in modulating masticatory function, may have offset the reduced proprioceptive input from the anesthetized muscle spindles^55^. This neurosensory compensation could account for the absence of performance decline despite sensory blockade. In addition, insufficient PMR-mediated compensation has been associated with impaired mastication in certain individuals^21,56^.

As expected, the participants reported higher fatigue levels after the chewing tasks with the hard candy and the two-coloured gum in both rounds, the scores were even higher in the second round. A statistically significant increase in fatigue after chewing the two-coloured gum was found in women after lidocaine injection, and comparisons between groups showed higher fatigue values for women for the two-coloured gum (T) after injection (*p* < 0.026). The repeated muscle activity required for these tasks could have lead to a decline in muscle performance, characterized by a decrease in maximal force or power production in response to contractile activity^57^, contributing to the observed reduction in the ability to finely divide the candy into particles. Likewise, it is important to understand that different factors interact on an individual basis to produce muscle fatigue and that the effects depend on the type and level of muscle activity and the host response^58^. This cumulative effect underscores the importance of considering muscle fatigue as a significant factor in studies examining masticatory performance, specifically in women. From a clinical standpoint, fatigue could account for patients’ subjective experience of an inability to chew as well as their actual impaired chewing performance.

There was no difference in the variance of hue when comparing pre- and post-injection values, suggesting that the local anesthetic injection did not affect the participants’ mixing ability. Although the comminution and mixing ability tests are generally believed to be positively correlated, some literature suggests that the ability to mix and knead a food bolus evaluates distinct aspects of mastication rather than directly reflecting masticatory performance^59,60^. Therefore, applying both tests may provide deeper insights into objective masticatory function^61^. Since mixing ability involves numerous structures (such as the teeth, palate, tongue, cheeks and neural pathways guiding the mandibular force and movements through muscle control)^38^, it is plausible that other structures adapted, maintaining this ability. This observation could be explained by the fact that the tongue, which was not affected by the anesthesia, plays a crucial role in the mixing of food. Its continued functionality likely compensated for any deficits in the masticatory muscles, ensuring the mixing process remained efficient despite the administration of local anesthetics. Furthermore, there is some evidence that larger values of masticatory performance are better assessed by the comminution test than by the mixing ability test. This may explain the absence of observed changes in values before and after injections^46^.

As the findings of the present study demonstrated reduced masticatory performance and lower efficiency in comminuting food after receiving injections of local anesthetics in women, it is relevant to highlight the gender differences in physiological mechanisms involved in mastication, which could have contributed to these results. Anatomical variations play a crucial role, studies have revealed that men exhibit greater muscle mass and higher muscle thickness compared to women^62,63^; as well as a greater bite force and a occlusal force, which also appear to be higher in men. These anatomical advantages likely contribute to more effective mastication in men. mastication^54,64^. Additionally, hormonal influences may modulate masticatory function. Estrogen seems to affect neuromuscular control and sensory perception^65^. To minimize hormonal variability across the menstrual cycle, all female participants in the present study were using hormonal contraceptives. This methodological approach reduces the potential confounding effects of fluctuating hormone levels on our results. Neuromuscular differences further contribute to the observed gender differences, as women exhibit longer chewing cycles than men. The literature supports that men have larger masticatory movements and shorter cycle times compared to women, facilitating a faster food breakdown^66,67^. This could explain the decreased efficiency observed in women in our results. Finally, neural mechanism related to sensory processing may also play a role. Evidence suggests that men show more pronounced changes in brain activation related to sensory integration and cognitive control areas during transitions between hunger and satiety. Such differences in neural activation patterns could potentially influence how sensory information from the oral cavity is integrated with motor commands during chewing^68^. These physiological factors provide a plausible explanation for the observed gender differences and are consistent with previous findings.

Finally, all these findings regarding food comminution are directly related to the method used for quantifying masticatory performance. Although traditional sieving methods using silicone-based test foods such as Optosil Comfort have been widely regarded as a reference standard, they are time-consuming and requires specialized laboratory equipment.^46,69–72^ In contrast, the optical scanning technique employed in this study offers a validated, time-efficient alternative for quantifying particle size distribution^46,73–75^. Beyond its operational advantages, this method has demonstrated strong agreement with sieving outcomes and exhibits high ability to detect interindividual variations in masticatory efficiency^73,76^. The present results, therefore, not only reflect meaningful physiological differences but are also supported by a reliable and reproducible measurement strategy grounded in previous methodological literature.

## Study strengths and limitations

This study offers methodological strengths that enhance the validity of its findings, to minimize the risk of confounding factors. The study involved a homogeneous matched group in terms of sex and age; and met strict inclusion criteria to ensure sample homogeneity. All participants were in good general health, with no comorbidities and not taking any medications. Questionnaires confirmed that this sample was representative of the study population and free from any conditions that could compromise the results. Regarding the test food, it was selected based on prior validation of their rheological behavior and used under standardized conditions (size, weight, storage, and chewing time); and were from the same batch. This ensured that all participants were exposed to identical conditions, and any potential effect of the candy would have been consistent across individuals, minimizing the risk of variability related to the test food. Another strenght of the study is the fixed test sequence for chewing tasks, structured to minimize fatigue and ensure reproducibility. Starting with non-anesthetized conditions and softer foods allowed participants to serve as their own controls under stable conditions, reducing potential order effects.

A possible limitation is the unintentional swallowing of candy fragments, assessing food comminution involves risks of unintentional swallowing and incomplete collection of particles, which could lead to measurement errors. This risk may be higher in S2 and H2, where a natural chewing pattern was used. Aditionally, the choice of test material can also affect the outcomes; only viscoelastic food was analyzed^21,28,39^. Different foods or food substitutes may present varying resistances and textures, which can influence chewing efficiency and, consequently, test results.

Another limitation of the study is the lack of objective verification of successful muscle spindle afferent blockade. Only a subjective analgesic manifestation was obtained from participants to confirm the anesthesia effect. However, the study employed a well-established clinical model involving lidocaine, which is widely used for muscle block procedures and has demonstrated effective clinical outcomes^42,77,78^. Local anesthetics have been applied to investigate the role of peripheral inputs in several clinical trials, and it is reasonable to presume the occurrence hypoalgesia/hypoesthesia following their administration, since peripheral mechanisms play a substantial role in sensory signs and symptoms^79^. Lidocaine effects extend beyond muscle spindle afferents, potentially influencing other sensory receptors and nerve fibers^80^. It can depress ectopic activity in A- and C-fibers and inhibit muscle-type nicotinic acetylcholine receptors, affecting neuromuscular transmission^81^. Despite these broader effects, lidocaine remains widely used due to its proven clinical efficacy. Although the precise extent of muscle spindle afferent blockade cannot be objectively confirmed, evidence from previous clinical trials with positive outcomes supports the assumption that this model effectively blocks a sufficient proportion of muscle afferents for experimental purposes^82^.

Future studies should assess how masticatory performance is affected by the altering of mechanoreceptors in the temporomandibular joint as well. This will help to further expand our understanding about the different mechanisms involved in the masticatory system. Indeed, it would also be interesting to conduct a similar study on patients with myalgia for comparison with a healthy population. Additionally, future studies need to test other type of food with different mechanical and rheological properties, or different sizes of the test bolus. These findings should be interpreted within the scope of the study’s design and sample.

## Conclusion

The altering of muscle spindle afferents by anesthetizing the jaw closing muscles appears to lead to a deficit in the masticatory function with reduced efficiency in comminuting viscoelastic food when compared to normal mastication in women. The mixing ability remained unaffected for all participants. This highlights the critical role of muscle spindle input in masticatory performance. The observed sex differences suggest that women may be more vulnerable to neuromuscular control changes following sensory alterations, emphasizing the need to consider these factors in the diagnosis and management of jaw-related conditions.

## Data Availability

The datasets used and/or analyzed during the current study are available from the corresponding author on reasonable request.
